# Challenging menstrual silence in sport: adolescent girls' perspectives on menstrual health, communication and change

**DOI:** 10.3389/fspor.2026.1862668

**Published:** 2026-10-30

**Authors:** Eva Åkerman, Emilia Åslund, Inger Eliasson, Rebecka Hallencruetz, Taru Tervo, Marie Klingberg-Allvin

**Affiliations:** 1 Department of Women’s and Children’s Health, Karolinska Institutet, Stockholm, Sweden; 2 Goteborgs Stad, Gothenburg, Sweden; 3 Department of Education, Umeå School of Sports Sciences, Umeå University, Sweden; 4 Floorball Research and Development Centre, Umeå School of Sports Sciences, Umeå University, Sweden

**Keywords:** adolsecent, communication, floorball training, menstrual health, menstrual management

## Abstract

**Background:**

Menstrual health has historically been a taboo subject and under-researched in sport. Although research on this topic is increasing, studies focusing on non-elite adolescent girls from a child rights perspective remain limited. Floorball, a male-dominated sport, is one of the most popular indoor team sports among adolescents in Sweden. This study explored the menstrual health experiences of non-elite adolescent girls playing floorball.

**Methods:**

Sixteen girls aged 12–18 years were interviewed. Data were analysed using reflexive thematic analysis.

**Results:**

Adolescents described physical discomfort, mood changes, fatigue and fear of menstrual leakage during menstruation, which sometimes reduced their performance in training and competition. Despite believing that menstruation should not be a taboo topic, menstrual discomfort was often managed in silence, and discussions about menstruation were limited due to embarrassment or perceived stigma, both among teammates and with coaches. Adolescents expressed openness to change and suggested several solutions for a more menstrual health-friendly environment: improved menstrual cycle knowledge among coaches, normalisation of menstrual conversations within the team and access to menstrual products at sports facilities. Coaches were identified as key actors for change, as adolescents expressed that knowledgeable and supportive coaches could help normalise menstruation and reduce stigma.

**Conclusions:**

Adolescents' perspectives on menstrual-related challenges and solutions are essential for developing menstrual health-friendly sports environments. Integrating menstrual health into coach education, supported by organisational policies and practical resources, may strengthen coaches' competence and confidence to communicate about menstruation and provide appropriate support to adolescent athletes.

## 1 Introduction

Menstrual health is defined as “a state of complete physical, mental and social well-being and not merely the absence of disease or infirmity, in relation to the menstrual cycle” (1). The menstrual cycle (MC) is the recurring physiological process involving cyclical hormonal changes. It begins on the first day of menstrual bleeding and ends on the day before the start of the next menstrual bleeding. According to Hennegan et al. (1), achieving good menstrual health requires fulfilment of five key components with access to the following: (i) accurate, timely and age-appropriate information about the MC throughout the life course; (ii) affordable menstrual products and supportive facilities, including water, sanitation, safe space for private use, cleaning and disposal of menstrual materials; (iii) timely diagnosis, treatment and care for menstrual discomforts and disorders, including access to health services, pain relief and self-care strategies; (iv) supportive social environment free from stigma, shame, or psychological distress; and (v) freedom to participate fully in all areas of life such as education, work, sports and social activities without facing exclusion, limitations or discrimination due to menstruation. In this study, our understanding of menstrual health is based on this holistic definition.

Despite increasing global recognition of menstrual health as a vital component of gender equality and human rights (2, 3), menstruation remains a stigmatised issue across diverse cultural and social contexts, including sport (4, 5). Research on the MC has primarily focused on elite female athletes and conditions related to the female athlete triad and low energy availability, as well as MC disorders such as oligomenorrhoea and amenorrhoea. A recent review consisting of 60 studies involving 6,380 female athletes demonstrated that the prevalence of MC symptoms and MC disorders are common and varied widely among female athletes (6). Dysmenorrhea is among the most frequently reported MC disorders (7–10). Furthermore, female athletes commonly experience negative MC-related symptoms during the premenstrual and menstruation phases, where affective symptoms are more prevalent than physical symptoms (9, 11).

Additional factors influencing participation and performance in sports include menstrual stigma and taboo (12). Menstrual stigma means having negative attitudes towards menstruation and people who menstruate, shaped by social norms and beliefs (13). Johnston-Robledo et al. described menstrual stigma as a socially constructed perception that positions menstruation as shameful, dirty and something that should be concealed (13).

The stigmatised status of menstruation can have profound consequences for girls' and women's self-esteem, body image and self-presentation. A review by Harvey et al. (14), including adolescents aged 10–18 years, identified menstrual stigma as one of several barriers to physical activity participation. Fear of menstrual blood leakage during sport has also been reported as a common concern among adolescents (11, 15), leading some girls to avoid physical activity during menstruation (16). Furthermore, fear of leakage has been associated with feelings of self-consciousness and reduced confidence during sports participation (15). Studies have also shown that menstrual stigma and taboo can hinder open communication about the menstrual cycle between young athletes and coaches (17).

The lack of open communication may also contribute to inadequate support for managing menstruation in sport. For example, athletes aged 16–18 years reported a range of physical and emotional symptoms throughout the MC that they felt affected their performance (18). They expressed a lack of support for menstrual management, with insufficient education and persistent stigma making it difficult to discuss menstrual-related concerns with coaches (18). Such communication was further influenced by factors including the coach's gender, age and coach–athlete relationship. Similarly, a study in a school setting showed that girls were reluctant to discuss menstruation with their physical education teachers, often making excuses to avoid participation and expressing concerns about unsuitable clothing (19). Other reported barriers to participation included concerns about the effectiveness of menstrual products (19) and difficulties of managing menstruation during training or competition (11).

In summary, MC-related symptoms are common among girls and women, and can significantly influence participation and performance in sport. While studies suggest that non-elite adolescent female athletes face challenges related to menstrual management, stigma, and insufficient support (15, 18), research examining how they experience and navigate menstruation and envision menstrual health-friendly environments in sports clubs remains limited. From a children's rights perspective, this knowledge gap is particularly important. Children's rights in sport, an essential aspect of human rights, remain underdeveloped both within sports organisations (39) and sport research (40). The perspectives of adolescents are crucial; understanding their lived experiences offers a child-centred lens that can inform efforts to foster greater inclusivity and equity in sport. This study is novel in that it targets non-elite adolescent girls' sporting experiences, capturing how young athletes encounter menstruation in relation to sport. These early perspectives are critical for shaping supportive practices from the outset, which may reduce stigma, normalise communication and prevent premature sport dropout.

The Swedish context provides a particularly relevant case, as floorball is one of the most practiced sports among children and adolescents. Despite its popularity, the sport remains male-dominated: approximately 65% of registered players are boys and 35% are girls; most coaches are men. Girls are also more likely than boys to discontinue organised sport, a trend evident in floorball as well (20). In order to safeguard the best interests of children and young girls under the age of 18 (21), a deeper understanding of the conditions surrounding menstrual health in sport is needed. Expanding knowledge in this area is therefore crucial not only for advancing sport science but also for promoting gender equality and fostering inclusive sporting environments.

This study aimed to explore the menstrual health experiences of adolescent girls playing floorball, focusing on MC-related symptoms, their impact on sporting performance, management strategies and communication about menstruation. This study also aimed to explore their thoughts on what changes are needed to develop a more menstrual health-friendly environment in floorball clubs.

## 2 Material and methods

### 2.1 Study design

We conducted a qualitative study using an inductive approach based on interviews with 16 girls aged 12–18 years. Qualitative study designs are appropriate for exploring people's perceptions and experiences, providing in-depth insights into their perspectives and the underlying factors that can inform future health-promoting efforts (22).

### 2.2 Recruitment

We used purposive sampling to recruit study participants who were female adolescent floorball players aged 12–18 years (22). Initially, recruitment was limited to the floorball district of Dalarna in Sweden, but was later expanded to include all floorball districts nationwide in order to reach more adolescent players. An information letter about the study was sent to the Swedish Floorball Federation, which forwarded the information to local floorball coaches. The coaches were asked to disseminate the information letter to eligible adolescents. The information letter contained contact details for the research team, which interested adolescents could use to sign up or answer any questions about the study. The information letter was also distributed to coaches and floorball players at the two largest floorball tournaments in Sweden: the Gothia Floorball Cup in Gothenburg and the Storvreta Cup in Uppsala. Information about the study was shared on social media. Furthermore, all coaches for teams between the ages of 12 and 18 in Gothenburg were contacted, first through email and later by phone with the information letter, asking the coaches to inform their players about the study.

### 2.3 Ethics approval and consent to participate

Ethics approval was obtained from the Swedish Ethical Review Authority (registration number 2023-03252-01, 2023-07178-02). Informed consent was obtained from all participants before their participation. For participants under 15 years of age, written consent was obtained from their parents. Before the interviews, the participants received both written and oral information about the purpose of the study. They were informed that participation was voluntary and that they could withdraw from the study at any time without providing a reason. They were also informed that their answers would remain confidential and that identifying information would be deleted from the transcripts. All the participants agreed to have the interviews recorded.

### 2.4 Data collection

Semi-structured interviews were used to gain an in-depth understanding of adolescent floorball players' experiences of menstruation and its impact on sports participation. Given the existing knowledge on the research topic area, a semi-structured approach was considered appropriate (23), as it enabled the exploration of predefined topics while allowing participants to describe their experiences in their own words and introduce unanticipated perspectives. Accordingly, the interview guide was developed by the research team based on the study aim and existing literature on menstrual health, adolescent athletes, and the impact of menstruation on sports participation. The guide was further informed by the menstrual health definition proposed by Hennegan et al., with questions designed to explore factors that support the achievement of menstrual health within the context of floorball participation. The semi-structured interview guide covered the following topics: experience of menarche, information and knowledge of menstruation and the MC, access to menstrual products in sports facilities, communication about menstruation in the floorball environment, menstrual symptoms and symptom management, experiences of healthcare and treatment related to menstrual concerns, the effect of the MC on general health and sporting performance and participation, menstrual management strategies in floorball environment, the impact of menstruation on daily life activities, and perceptions of a menstrual health-friendly floorball environment. The interview guide was pilot tested with two adolescent floorball players and revised to improve clarity. Following the first interview, minor revisions were made to the interview guide.

In total, 16 individual interviews were conducted from November 2023 to June 2024. Three researchers (EÅ1, EÅ2 and RH) conducted the interviews. Having multiple interviewers provided flexibility in scheduling interviews according to participants' preferences and availability, while also facilitating data collection within the study timeframe.

To promote consistency across interviews, all interviewers used the same semi-structured interview guide. EÅ1 is a public health researcher who primarily focuses on menstrual health and has extensive experience conducting qualitative research. Prior to data collection, EÅ trained the interviewers (EÅ2, RH) in qualitative interviewing techniques and the use of the interview guide. All interviewers had substantial expertise in menstrual health, enabling informed and sensitive exploration of the study topics. RH has over 10 years of experience working with menstrual health and menstrual rights within civil society. EÅ2 holds a bachelor's degree in gender studies; her master's thesis includes menstrual health.

The adolescents could choose whether to be interviewed digitally through Microsoft Teams or in-person meetings. In total, 12 interviews were held through Microsoft Teams and four were held in person. The interviews lasted for 20–50 min. In total, 12 interviews were conducted via Microsoft Teams and four were conducted in person. In-person interviews were audio-recorded. Interviews conducted via Microsoft Teams were recorded using the platform's built-in recording function, which captured both audio and video, as audio-only recording was not available in Microsoft Teams at the time of data collection. Transcripts were anonymised and, together with the recordings, stored on a two-factor authentication-encrypted drive accessible only to the research team. Table 1 presents an overview of the adolescents' characteristics, including age, menarche, contraceptive use, and length of floorball participation.

**Table 1:** Respondents' characteristics.

Age group	*N* (has gone through menarche)
12–14 years	7 (6)
15–18 years	9 (9)
Menarche	Mean (Range)
Year	12 (10–13.5)
Using contraceptives	
No	12
Yes	4
Year practising floorball	
1–3	1
4–6	6
More than 7 years	9

### 2.5 Analysis

The data were analysed using reflexive thematic analysis, as described by Braun and Clarke (24). The thematic analysis aimed to identify and present themes in the participants' stories. The data were transcribed verbatim using f4p audio transcription software. Researcher EÅ2 reviewed both the audio recordings and the transcribed interviews to check for any typing errors and then corrected them.

Six researchers (EÅ1, EÅ2, IE, TT, RH, MKA) with different experience, competencies and professions participated in the analysis. Their combined expertise in menstrual health (EÅ1, EÅ2, RH, MKA), public health (EÅ), gender studies (RH, EÅ2), sport pedagogy (IE), sports medicine (TT), reproductive health (MKA) and children's rights in sports (IE) contributed to interpreting the data from multiple perspectives. Researchers EÅ1 and EÅ2 conducted the initial coding, while all members of the research team contributed to the development, refinement, and interpretation of themes throughout the analytical process. During the initial coding phase, researchers EÅ1 and EÅ2 identified various types of codes (e.g., value, emotion, invivo and process) to capture the participants' narratives as comprehensively as possible based on the study's purpose and the research questions (25). All the transcribed interviews were coded by at least two researchers (EÅ1, EÅ2). The codes were inserted directly in the right-hand margin of the transcripts, discussed by EÅ1 and EÅ2 and then adjusted and defined. Related codes explaining similar patterns were collated to form preliminary themes and subthemes. These were reviewed in relation to the coded extracts and the full dataset to ensure that they accurately reflected the participants' narratives and captured meaningful differences between themes. Themes were then refined, defined, and named through iterative discussions among all members of the research team.

## 3 Results

Through the analytical process three themes and seven subthemes were created, as illustrated in Figure 1.

**Figure 1:**
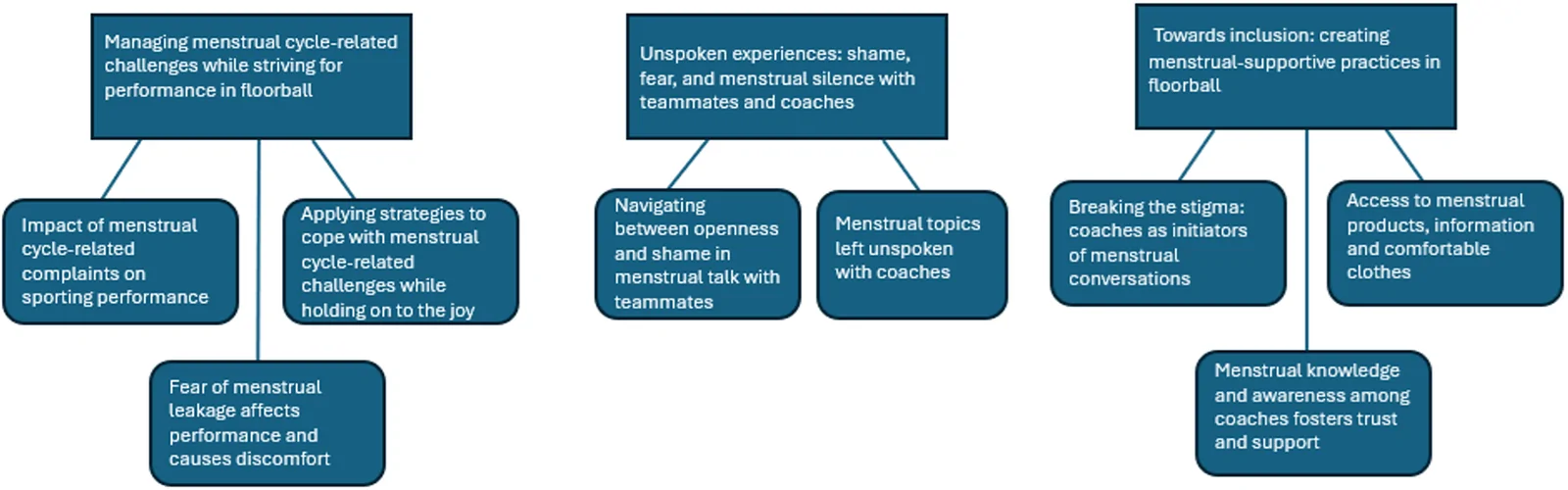
Overview of themes (square) and subthemes (rounded square) describing adolescents' menstrual-cycle challenges and supportive practices in floorball.

The names given for each quotation below are pseudonyms to maintain confidentiality.

### 3.1 Theme 1: managing MC-related challenges while striving for performance in floorball

Theme 1 describes the adolescents' perspectives and experiences of MC-related symptoms and challenges in relation to their participation in floorball. The enjoyment of playing floorball motivated them to find strategies that enabled continued participation, despite discomfort. Adolescents often adopted various menstrual management strategies to ensure that menstruation did not hinder their performance in floorball or daily activities.

#### 3.1.1 Impact of MC-related complaints on sporting performance

Almost all adolescents described experiencing symptoms of menstruation, although to varying degrees. The most common complaints mentioned were mood swings or feeling irritated and annoyed. In addition, experiencing menstrual pain was reported to negatively affect their mood. Several adolescents described how their menstruation and mood swings affected their floorball practice. One adolescent described her mood changes as one of her biggest issues:

It's a bit hard to train while menstruating. I don't know, it's just… just having to train while dealing with it. And it's exhausting, and often you don't feel so good, and it usually ends up with you feeling down and stuff. Then you don't really feel motivated to go to practice. Or to school. Or to do anything at all, really. (Olivia, 15 years old)

Another complaint was menstrual pain, which was described as having a greater impact on participation. Fanny, who had severe menstrual pain and other discomfort related to menstruation, expressed the following:

I often have a lot of pain in my stomach specifically, and I frequently experience headaches and feel dizzy and faint. Mostly it's easier to feel dizzy and faint, especially when I am training which makes me passive and cautious. (Fanny, 13 years old)

Despite not feeling well during her menstruation, Fanny has skipped floorball practice only a few times. Most of the adolescents stated that they usually do not skip practice or any other activity while acknowledging that it has happened once or twice. The adolescents described how they went to practice as usual, no matter if they were bleeding and experiencing menstrual pain and/or other complaints. A few of them used expressions such as “*you've just got to do it*” (Pia, 16 years old); *“I wouldn't want it [menstruation] to stop me really”* (Klara, 16 years old).

However, due to menstruation, several adolescents reported not being able to perform at their full capacity, noticing that they ran slower and felt more irritable or sensitive during practice. “*For me, at least, when I get my period, I can't give my full effort because sometimes I experience a lot of pain”* (Lisa, 15 years old). Running with cramps was described by some as uncomfortable and unpleasant, yet many adolescents found practice enjoyable despite the pain. Some warm-ups were found to be harder to perform when menstruating, while practice was described as a distraction from menstruation and cramps.

#### 3.1.2 Applying strategies to cope with menstrual cycle-related challenges while holding on to the joy

When answering further questions, some adolescents described different ways of adjusting or employing strategies to manage their menstrual complaints during practice. Their enjoyment of playing floorball motivated them to find strategies that allowed them to continue participating despite discomfort. The experiences of menstruation varied among the adolescents, which was also reflected in different strategies for participation and the extent to which menstruation affected their sporting performance.

Although adjusting or skipping practice was said to be undesirable and rare, the adolescents described their own strategies and adjustments to participate and perform as usual during practice and gameplay (Table 2). Those with severe symptoms, such as cramps and faintness, preferred to take it easier. If heavily affected by their menstruation, some described practising shooting instead of running: “*I'll just stand and shoot instead. I don't have the energy to run right then*” (Olivia, 15 years old). Resting a little bit extra, doing their own version of warm-up, and not doing all the movements were other strategies. “*I try to avoid certain positions if they hurt more, and take it a bit easier, rest a bit more. Sometimes when I push myself too hard, I get completely exhausted. So I try to adjust my training based on how I feel*”*.* (Lisa, 15 years old)

**Table 2:** Management strategies.

Management strategies
Taking painkillers and seeking medical advice Taking it easier and some extra rest Doing their own version of warm-up exercises

Taking painkillers before and during practice was common among the adolescents to relieve cramps. Doing other exercises was also mentioned as a strategy to manage menstrual pain. Some also reported that training had no effect on menstrual pain. “*I've heard that exercise can help with menstrual pain. But personally, I don't think it has worked that well for me*” (Klara, 16 years old)*.* A few described a tendency to forget menstruation as they focused on playing floorball and “just going for it”. “*Then, I don't really care… if I have my period or not […] then I just take double Alvedon [Paracetamol], if I'm really in pain.*” (Olivia, 15 years old)

A few used contraceptives to either remove their menstruation or to ease their pain and other complaints. Ellie, 15 years old, described her mood changes and severe menstrual pain as one of her biggest issues, which made her turn to medical professionals as the pain made her feel very angry: “*I was kind of really angry and had a lot of pain in my stomach. I could withdraw into myself, sort of shut myself in my room.*” (Ellie, 15 years old)

When the adolescents were asked if they would like to adjust their floorball practice due to the degree of menstrual complaints, most stated that they would not want to adjust their practice. The reasons for this were the fear of not being prepared for a game, simply not wanting menstruation to stop them and that adjusting might affect the whole team.

#### 3.1.3 Fear of menstrual leakage affecting performance and causing discomfort

Worry about leaking while menstruating during floorball practice and matches was described as affecting the performance of some of the adolescents. A few also described how fear of leaking affected their ability to concentrate on practice. “*Yes, well, it would be a nightmare to leak […] you should not have to feel ashamed about it, but I think I would have anyway*” (Johanna, 16 years old). Two adolescents described how they wear a pair of extra shorts underneath their regular clothes during practice as a strategy to avoid leaking, feel safer and prevent it from distract them while playing floorball.

Challenges related to menstrual blood also included fear of others finding out that they were menstruating during floorball practice or matches. Isabelle, 12 years old, felt worried about the possibility that someone might notice she was wearing pads.

When I change clothes, I change pretty quickly because I'm afraid someone will see that I'm wearing, like, a pad. So that’s it, changing clothes and showering can be a bit, uh, uncomfortable, especially when I'm on my period. (Isabelle, 12 years old)

Despite feeling safe with her teammates and believing that her teammates would not say anything about her period, Isabelle adopted strategies to avoid others noticing that she was menstruating. She described how she would take her shower after her teammates had finished and never shower at the same time.

### 3.2 Theme 2: unspoken experiences: shame, fear, and menstrual silence with teammates and coaches

Theme 2 refers to the stigma, fear and silence surrounding menstruation, which were clearly reflected in the adolescents' narratives within the floorball setting. Although they believed that no one should feel ashamed to talk about menstruation, these experiences often remained unspoken with their teammates and coaches, despite menstrual complaints that negatively impacted their floorball practice. None of the adolescents used the word “stigma”; instead, some described discussing menstrual issues openly as somewhat embarrassing and expressed a fear of being discovered while playing floorball during their period.

#### 3.2.1 Navigating between openness and shame in menstrual talk with teammates

The adolescents were asked to reflect on whether they talked about menstruation with their teammates. Most perceived that they had an open environment with their floorball teammates where they could talk about menstruation if they needed to. However, most of them said that they do not talk about menstruation often with their teammates. This was commonly apparent among all the younger participants between the ages of 12 and 14, who reported that they did not talk about menstruation with their teammates. “*We don’t really talk about it that much. (Anna, 12 years old) [Interviewer: Has it ever happened that you have talked about it?] No, we have not.*” (Anna, 12 years old)

Not talking about menstruation due to embarrassment or shame was mentioned by several of the younger adolescents. “*I think a lot of people find it embarrassing to talk about.* [*Interviewer: Why do you think people feel that way?] Uh, well, because you bleed from down there (laughs).*” (Cecilia, 13 years old) Another adolescent described menstruation as awkward or embarrassing, which is why it is not brought up during practice. Others mentioned that not everyone on the team menstruates yet. In contrast, one older adolescent shared her view on talking about menstruation:

I think we're pretty open about almost everything, like… people don't really get embarrassed… someone might just say, ‘ugh I got my period yesterday, I don’t feel like training’. (Olivia, 15 years old)

Some explained that they do not feel the need to talk about it because they have no menstrual issues. Some adolescents said they simply do not think much about menstruation, while one admitted that she does not know why it is not discussed among teammates. On one team, it was noted that practice was the only time they met, as they attended different schools, so they preferred to talk about other topics. Others felt it was unnecessary since their team members were older and already knew what they felt they needed to know about menstruation.

Well, I don't know, there are many who think it is a little hard to talk about. […] but then there are those in my team who are very open to talking about such things… I guess that it's just something you would rather not talk about. (Hanna, 17 years old)

A few adolescents reported examples of how they talked openly with their teammates about menstruation. For example, they could ask each other for menstrual products when needed and inform each other when bleeding to explain how they feel and to receive empathy, since menstruation during practice can be hard for some. Some adolescents stated that they wanted to talk more about menstruation within their teams. Several expressed the need for and desire to have an open environment for discussing menstruation. They believed this would make it easier to talk about menstruation, helping them feel safer in the sport context, and also contributing to normalising such conversations. A few adolescents also explained that talking about menstruation and sharing experiences could be a helpful way to be better prepared for their own menstruation.

[…] I don't know, it might feel good for some, who maybe not have had it much or do not have it to hear about it more. It might be relaxing for some and maybe hard for others […]. It could be hard for people who have not gotten it and then you might feel that you are the only one who has not gotten it yet […] But I think it would feel, well good. For everyone. (Isabelle, 12 years old)

#### 3.2.2 Menstrual topics left unspoken with coaches

Adolescents were asked whether they had conversations about menstruation with their coaches. Most reported that they rarely discussed menstruation with their coaches, except when they need to ask for menstrual products or inform them about cramps and premenstrual syndrome (PMS). They emphasised that no one should feel ashamed to ask for menstrual products or to mention that they menstruate. A few mentioned that they would feel comfortable talking to their coaches about menstruation if necessary. However, they had not done so because they have not encountered enough issues with menstruation to warrant such conversations. “*Yes, I can do it [talk to coaches]. But I feel, it's like, right now I don't feel like I have any issues. But… eh, and if I did feel like I had any issues, then I would easily do it.*” (Gianna, 14 years old)

One reason why several adolescents did not talk to their coaches about menstruation was due to feelings of embarrassment. A few adolescents stated that their parents were one of their coaches, which made talking about menstruation easier. When the adolescents described not usually talking about menstruation with their coaches, some also stated that they do inform the coaches about their mood swings (PMS), if they experience menstrual pain during practice and if they need menstrual products.*“[…] if I hadn't brought it [menstrual products] myself, then I would go to… one of our coaches and say that I need it or just go and take it where I know it is and then go to the bathroom.”* (Hanna, 17 years old)

### 3.3 Theme 3: towards inclusion—creating menstrual-supportive practices in floorball

Theme 3 relates to adolescents' suggestions for improving the floorball environment and supportive practices during menstruating. Adolescents' suggestions emphasised that coaches play a key role in breaking stigma by initiating menstrual health conversations and increasing their menstrual knowledge to better support players**.** They also expressed a need for access to menstrual products, information and comfortable clothing to feel safe and supported.

### 3.4 Breaking the stigma and shame: coaches as initiators of menstrual communication

Most of the adolescents stated that their coaches had not initiated talking about menstruation with them or their teams during practice. The coaches who had brought up the topic of menstruation with the teams had mostly informed the adolescents about where they could find menstrual products, which was described as helpful.

All adolescents expressed positive views of coaches who take the initiative to discuss and provide information about menstruation. However, they acknowledged that it might feel unfamiliar for both the coaches and the adolescents. Isabelle, 12 years old, expressed:

I think it could be good, as I said, for people who haven't experienced it to learn a bit more about it, but I think it would create a pretty awkward atmosphere in our team [laughs]…We only have male coaches, so it would probably be a bit awkward if they started talking about, you know, menstruation and stuff like that.

Even those who said they did not to need menstrual-related conversations were positive about such conversations. They believed that fostering an open environment and providing information about menstruation would help reduce feelings of embarrassment and shame.

Several adolescents mentioned that it would be beneficial if coaches brought up the topic, especially for younger players and those who found discussing menstruation embarrassing or difficult. A few adolescents noted that while discussing menstruation might initially feel uncomfortable and awkward, they recognised that such conversations could benefit the entire team. Some stated that openness would create a safer environment and encourage team members to approach coaches about the topic if needed.

Maybe, or at least so that the whole team becomes more comfortable talking about it, because I don't know if everyone is […] actually, you should be able to share how you are feeling and stuff like that. (Klara, 16 years old)

Even though menstrual conversations with male coaches could be embarrassing, all adolescents were positive about male coaches initiating communication about menstrual topics. “*…if it is a female coach then it's like, well she knows how it is and she is a support, she has been through it. If it's a male coach […] but, it often also feels good when they do it, because it… shows that they actually care.*” (Olivia, 15 years old)

By educating coaches on menstrual health, adolescents suggested that this could equip them to initiate conversations about menstruation—and help them be perceived as more serious and supportive.

### 3.5 Menstrual knowledge among coaches fosters trust and support

The adolescents' narratives revealed that coaches' knowledge of MC-related symptoms fosters trust and support. For example, nearly all expressed that it feels easier to obtain information and have menstrual conversations with a woman, since she has her own experience of menstruation and therefore a deeper understanding of the topic. “*Maybe female [coach] because it feels like men do not really know what they are talking about.*” (Ellie, 15 years old) Adolescents perceived that male coaches' lack of personal experience with menstruation was one reason for their limited knowledge and understanding. One of the adolescents mentioned a lack of knowledge about mood swings and variations in bleeding:

Well, mood swings … because I don’t think they know much about it, PMS, […] that you bleed a lot and that it is different for everyone. That it's not the same for everyone and some have more pain. Some barely notices. So that they have a better understanding. (Lisa, 15 years old)

In general, the adolescents wished that coaches knew more about how much menstruation varies between individuals regarding both physical symptoms and how it can affect the mood of the adolescents during practice. Beatrice, 12 years old, stated, “*I think you kind of need to know everything about it, like almost everything*”. Another adolescent expressed: “*Well, it would be good if they know as much as possible […] how you can feel, and obviously it differs from person to person, but, how it often can be and what you might need.*” (Johanna, 16 years old)

Furthermore, some adolescents said that the coaches do not fully understand that athletes sometimes need to rest and take things more slowly due to menstrual pain. There were also examples of how coaches can create supportive situations through their understanding of menstrual cycle-related symptoms. One adolescent described that her coach once bought chocolate for the team when several of them experienced PMS at the same time during practice. Having chocolate allowed them some time to rest, which was appreciated.

You can tell when someone has like, PMS, and there was this one time when a bunch of us were feeling that way, and our coaches went and grabbed a bunch of chocolate from the kiosk (Johanna, 16 years old).

One adolescent described having a good relationship with her coaches because they listened to her; this made her feel comfortable talking to them about most things regarding menstruation. An enhanced understanding of menstruation among coaches was identified as one way to better prepare them for the various symptoms associated with menstruation, thereby enabling more effective support for the adolescents. The desired support, as described by the adolescents, included emotional aspects, such as modifying the way coaches communicate, offering encouragement rather than pressure and inquiring about the adolescents' feelings and needs. “*Make sure that they have everything they need, check if they feel alright, maybe a little extra.*” (Fanny, 13 years old)

One thing which would be better I guess is that you can talk more about it. […] that we who play should talk, or talk about it […] that you have an open dialogue and that everyone talks. (Denice, 15 years old)

Some adolescents also stated that it would be helpful if the coaches said more positive things, to have some extra patience and give them pep-talks to make it easier for them to participate during menstruation.

I think a bit more accommodating anyway, a little more understanding but still try to cheer them on that they can continue […]and try to get through the game but not pressure them too much in case someone has a lot of pain. […] Let them take it a little easier if there is a game, that you should try, and make them try their best. (Lisa, 15 years old)

### 3.6 Wish to have access to menstrual products and comfortable dark clothes

All adolescents stated that floorball clubs and their coaches should provide menstrual products. This includes both tampons and pads in the locker rooms, in the first aid kits and in bathrooms. “*…have menstrual products in bathrooms and purchase menstrual products, so you can have them in the first aid bag and such.*” (Anna, 12 years old)

Black or dark clothes, specifically shorts, to play in during practice and matches were described by most of the adolescents as making them feel safer and not worrying as much about leaking blood. Most of the adolescents said they wore dark or black clothes when playing floorball. It was also stated that if you were to leak, it would not be as stressful or embarrassing for the adolescents because it would be less noticeable.

## 4 Discussion

Our findings highlight that the adolescents encountered various challenges when playing floorball due to their MC-related symptoms. Adolescents employed different strategies to manage their MC complaints during practice or adjusted their floorball practice since they were unable to perform at their full capacity. These challenges and coping strategies were often managed in silence, as many adolescents did not openly discuss their experiences with either coaches or teammates despite believing that menstruation should not be a taboo topic. Importantly, this unique study provides insights into their suggestions for developing a menstrual health-friendly environment for floorball. Adolescents expressed a desire for change and proposed several solutions to promote a menstrual health–inclusive environment within floorball. These included enhancing coaches' knowledge of the MC and encouraging them to initiate conversations about menstruation to create an open environment.

Consistent with previous research on athletes' experiences with MC in sport (6, 18, 26), the adolescents in this study reported experiencing dysmenorrhea, mood changes and other symptoms. Previous studies have shown that MC-related symptoms such as dysmenorrhea, fatigue and mood disturbances can impair sporting performance, disrupt participation and influence both training and competition (14, 26, 27). Similarly, the adolescents in our study reported that they experienced a range of symptoms that limited their ability to perform at full capacity. The severity of menstrual symptoms varied among the adolescents, as did their impact on performance. Enjoying floorball was a key reason why some adolescents remained active during menstruation despite experiencing severe symptoms. Consistent with previous research (19), some adolescents reported that engaging in training helped distract them from their menstrual symptoms.

The strategies employed by adolescents to manage menstrual complaints—modifying training intensity, using pain relief or adjusting practice schedules—reflect a high degree of self-management and resilience. Notably, these coping strategies are frequently managed in silence, with limited open discussion among peers or coaches, despite a shared belief that menstruation should not be a taboo subject. The tendency for adolescents to manage menstrual complaints in silence aligns with previous research demonstrating that menstruation remains a stigmatised and taboo topic in sports settings, hindering open discussion about menstruation (17, 18, 28–30). Similar patterns have also been documented beyond sport settings, where menstrual symptoms are often managed privately and in silence, reflecting broader social norms and behavioural expectations to conceal menstruation (31, 32). Behavioural expectations to conceal menstruation may also discourage individuals from seeking support despite experiencing menstrual health needs (33). According to Johnston-Robledo and Chrisler (41) menstruation can be conceptualised as an invisible social stigma. Menstrual blood is often regarded as dirty or unclean, making it a stigmatising attribute that menstruating individuals are expected to hide or manage discreetly (31). This broader conceptualisation of menstruation as a socially stigmatised bodily process may help explain why participants in the present study largely manage symptoms on their own and reported limited communication about menstruation, despite expressing that menstruation should not be a taboo subject. The menstrual stigma was also reflected in the management strategies described by the study participants, such as wearing extra pants, preferring dark pants or avoiding showering at the same time as others while menstruating. Consistent with previous research (18), this study's participants expressed fear of menstrual leakage and the possibility that others might notice visible signs of menstruation. Worrying about leaking affected their ability to concentrate during floorball training and matches, which in turn affected their performance. These experiences suggest that menstrual stigma may not only shape self-management practices, but also directly impact athletic performance by drawing cognitive and emotional resources away from the sport performance itself. Similar findings have been reported in the broader menstrual health literature, where concerns about concealing menstruation and avoiding visible signs of menstruation have been linked to increased mental burden and emotional distress (31).

Study participants proposed concrete solutions to receive better support from coaches and address the silence around menstruation, such as improving coaches' knowledge of menstruation and encouraging them to initiate open conversations. These adolescent perspectives are complemented by a recent Swedish study on floorball coaches of adolescent girls, showing that coaches experience communicative, educational and organisational barriers when addressing menstrual health, while also expressing a need for increased knowledge and structured guidance (34). Together, these findings align with a growing body of research emphasising the need to support coaches by providing education on the MC (18, 35, 36) and practical guidance on how to support players' health and well-being (36). Improved confidence in MC communication can help players feel supported and can contribute to normalising conversations about the MC (36). This was evident in our study, where adolescents expressed that greater confidence in MC communication among coaches can foster support and help normalise such conversations. Implementing menstrual health education within coaching practices may not only help to normalise MC communication but also has the potential to enhance adolescent athletes' well-being, performance and overall sporting experience (15, 26). Adolescents' positive perceptions of coaches initiating conversations about menstruation and their views that menstruation should not be a taboo topic demonstrate their openness to change.

### 4.1 Implications of the findings

From a child rights perspective, the voices of adolescents themselves, expressing both the challenges they face that affect their sporting performance and the solutions they propose, should inform the development of efforts to create menstrual health-friendly sports environments. Creating such environments may be supported by strengthening coaches' knowledge of the MC and its potential impact on adolescent athletes' participation and performance. Integrating menstrual health into coach education, supported by organisational policies and practical resources, is essential to strengthening coaches' competence and confidence. Such efforts can break the cycle of stigma, normalise MC communication, support adolescent athletes' well-being and performance and advance gender equity in sport. Further research and practice-based efforts are needed to explore how menstrual health education can be implemented within the floorball context and what factors may facilitate this process**.** Furthermore, research should be gender- and age-sensitive, focusing on how coach education can accommodate both male and female coaches and meet the needs of players at different developmental stages, including pre- and post-menarche.

### 4.2 Strengths and limitations

A notable strength of this study is the involvement of a multidisciplinary team in the analysis, consisting of six researchers with diverse professional expertise in physiotherapy, sports medicine, midwifery, sport pedagogy, reproductive health, gender equality and public health. This form of investigator triangulation enabled the data to be examined from multiple perspectives based on significant areas of expertise, thereby enriching both the analysis and interpretation and strengthening the study's credibility (37). Credibility and transparency were further supported through the use of participant quotations. We also sought to strengthen dependability by providing a clear and detailed account of the research process, including data collection, analysis and the presentation of results (37).

However, certain limitations must be acknowledged with respect to the transferability of the findings—the extent to which the results may be applied to other populations and contexts (38). While the interviews generated rich and nuanced data, the relatively small sample means that the findings should be interpreted as reflecting the experiences of this specific group of participants rather than adolescent floorball players more broadly. Further, participants were not asked whether they identified as transgender or non-binary, nor were data collected on self-reported experiences of disability—factors that may influence perspectives related to menstrual health (1). This limits the transferability of the findings to transgender, non-binary and disabled individuals. Future research should include adolescents with trans and non-binary identities, as well as those with lived experiences of disability; such perspectives may provide valuable insights into how more inclusive menstrual health environments in sport can be fostered.

In addition, this study was limited to adolescents with experience in floorball, which may restrict the transferability of the findings to other sports. Nonetheless, several findings on menstrual taboo and stigma in relation to communication were similar to those reported among young athletes in other countries. Finally, experiences of menstrual stigma and taboo do not appear to be bound to a particular context or nation, although their manifestations may differ across settings.

## 5 Conclusions

Adolescent floorball players reported several menstrual-related complaints that affected their training and competition. Despite these challenges, many continued to play floorball during menstruation, demonstrating commitment, resilience and self-management. Most managed menstrual complaints independently and in silence, without discussing their experiences with their coaches or teammates. These findings demonstrate the persistent stigma and discomfort surrounding menstruation in sport, limiting open communication which may hinder young athletes from receiving the support they need. Adolescents identified coaches as key actors for change, emphasising that knowledgeable and supportive coaches could help normalise menstruation and reduce stigma. Integrating menstrual health into coach education, supported by organisational policies and practical resources, may contribute to more menstrual health-friendly sports environments.

## Data Availability

The datasets presented in this article are not readily available. The datasets are subject to access restrictions because participants were not informed that the data might be shared beyond those involved in the study. To respect participant expectations and ethical standards, access to the datasets should be limited to researchers directly involved in the study. Requests to access the datasets should be directed to eva.akerman.2@ki.se.
